# Curcumin anti‐tumor effects on endometrial cancer with focus on its molecular targets

**DOI:** 10.1186/s12935-021-01832-z

**Published:** 2021-02-18

**Authors:** Fahime Jahanbakhshi, Parisa Maleki Dana, Bita Badehnoosh, Bahman Yousefi, Mohammad Ali Mansournia, Moghadeseh Jahanshahi, Zatollah Asemi, Jamal Halajzadeh

**Affiliations:** 1 Department of Gynecology and Obstetrics, School of Medicine, Shahid Beheshti University of Medical Sciences, Tehran, Iran; 2 Research Center for Biochemistry and Nutrition in Metabolic Diseases, Institute for Basic Sciences, Kashan University of Medical Sciences, Kashan, Islamic Republic of Iran; 3 Department of Gynecology and Obstetrics, Dietary Supplements and Probiotic Research Center, Alborz University of Medical Sciences, Karaj, Iran; 4 Molecular Medicine Research Center, Tabriz University of Medical Sciences, Tabriz, Iran; 5 Department of Biochemistry and Clinical Laboratories, Faculty of Medicine, Tabriz University of Medical Sciences, Tabriz, Iran; 6 Department of Epidemiology and Biostatistics, School of Public Health, Tehran University of Medical Sciences, Tehran, Iran; 7 Clinical Research Development Center (CRDC), Sayad Shirazi Hospital, Golestan University of Medical Sciences, Gorgan, Iran; 8 Department of Biochemistry and Nutrition, Research Center for Evidence-Based Health Management, Maragheh University of Medical Sciences, Maragheh, Iran

**Keywords:** Curcumin, Endometrial cancer, Apoptosis, Migration, microRNA, Inflammation

## Abstract

Curcumin is extracted from turmeric and shows a variety of properties that make it a useful agent for treating diseases and targeting different biological mechanisms, including apoptosis, angiogenesis, inflammation, and oxidative stress. This phenolic compound is safe even at high doses. However, it has poor bioavailability. The incidence rates of endometrial cancer (EC) that is one of the most prevalent gynecological malignancies is increasing. Meanwhile, the onset age of EC has been decreased in past few years. Besides, EC does not show a convenient prognosis, particularly at advanced stages. Based on this information, discovering new approaches or enhancing the available ones is required to provide better care for EC patients. In this review, we cover studies concerned with the anti-tumor effects of curcumin on EC. We focus on molecular mechanisms that are targeted by curcumin treatment in different processes of cancer development and progression, such as apoptosis, inflammation, and migration. Furthermore, we present the role of curcumin in targeting some microRNAs (miRNAs) that may play a role in EC.

## Introduction

Curcumin is a phenolic antioxidant extracted from turmeric, which is frequently used as a spice and has a yellow color [1, 2]. The rhizome of the herb *Curcuma longa* is the origin of turmeric that contains turmerin protein as well as analogs of curcumin, demethoxycucumin, and bisdemethoxycurcumin. 1,7-bis(4-hydroxy-3- methoxyphenyl)-1,6-heptadiene-3,5-dione is the chemical name of curcumin and C_21_H_20_O_6_ is its empirical formula [3, 4]. Since curcumin and its two analogs have the same molecular and biological characteristics, it is suggested that bisdemethoxycurcumin converts to demethoxycucumin, which in turn, transforms into curcumin. While curcumin plays a variety of beneficial roles, studies on animals and humans have concluded that it is a safe agent even at high doses [5]. However, a poor bioavailability has been attributed to curcumin [6, 7]. 1 to 2 h after consuming a single 4000 mg or higher oral dose of curcumin, its peak of plasma concentration can be observed [6]. Curcumin acts as an anti-oxidative, anti-microbial, anti-malarial, anti-HIV, and anti-angiogenic agent. Furthermore, it can be used in the treatment of inflammation, skin wounds, and neurodegenerative diseases [8–10].

Endometrial cancer (EC) is one of the most prevalent gynecological malignancies all over the world and its incidence rates are increasing [11, 12]. The mortality rate of this disease is growing more in older women than young women [11]. Moreover, the onset age of EC has been decreased compared to the past years [11]. EC can be categorized into two types: Type I and Type II [13]. Each one of these types exhibits some unique mutational profiles and clinical features. Primarily, tumors with histological characteristics of endometroid are considered as Type I tumors; whereas, Type II tumors show non-endometroid histological features. Loss of PTEN has been reported in 83 and 11% of Type I and Type II tumors, respectively. TP53 mutations have been reported in 10–20 and 90% of Type I and Type II tumors, respectively [14]. Mutations of PIK3CA have been found in 20–40% of Type I tumors, which is a higher number compared to 20% in Type II tumors. However, amplifications of this gene occur in 15% of Type I tumors, which is a lower number compared to 50% in Type II tumors. Furthermore, PIK3R1 mutations are more common in Type I tumors (43%) compared to Type II tumors (12%) [15].

The majority of patients with EC have abnormal uterine bleeding at the beginning of their disease. Endometrial biopsy or operative dilation and curettage (D&C) is being used for diagnosis, and in 99% of the cases, these methods lead to a histopathologic diagnosis [16]. Transvaginal ultrasound is another diagnostic tool [17]. EC prognosis is associated with multiple factors, such as histological subtype, grade, and the disease stage [18]. Besides, the survival of the patients and lymph node metastasis are significantly correlated with histological grade and the depth of myometrial invasion [19]. Studies also have shown several biomarkers that can be useful in predicting the outcome of the disease, including serum amyloid A, CA-125, CA 15 −3, CA 19 − 9, survivin, c-erbB2, cyclooxygenase, and L1 cell adhesion molecule [20]. Older age, family history of EC, early menarche, late menopause, obesity, exposure to radiation, and infertility are some of the risk factors of EC [21]. The chance of developing EC is lower in African or Asian women than in Caucasians [21]. However, white women have a better prognosis than black women at the same stage of the disease [22]. 28% of EC cases show regional or distant metastasis despite the early-stage diagnosis in more than 70% of the cases [23]. Particularly at advanced stages, this disease does not show a convenient prognosis [23]. There are different treatment options for advanced and recurrent EC, such as chemotherapy, radiation, hormone therapy, and surgery. However, common treatments are not capable of enhancing overall survival rates [24]. Also, none of the therapeutic methods is helpful in 15% of women with an aggressive phenotype of EC [25]. Thus, discovering new approaches or enhancing the available ones is required to provide better care for EC patients. In this review, we cover studies concerned with the anti-tumor effects of curcumin on EC. We focus on molecular mechanisms that are targeted by curcumin treatment in different processes of cancer development and progression, such as apoptosis, inflammation, and migration. Furthermore, we present the role of curcumin in targeting some microRNAs (miRNAs) that may play a role in EC.

## Curcumin and cancer

Since curcumin has interactions with several intracellular and extracellular molecules that are involved in various cancers, it is a potential candidate for suppressing cancer progression [26]. Curcumin has negative effects on processes involved in cancer, including apoptosis, angiogenesis, inflammation, and oxidative stress. Thus, it can serve as a beneficial agent in prevention, treatment, and controlling the symptoms of cancers, such as breast cancer, colorectal cancer, prostate cancer, melanoma, and lung cancer [26–32]. The complicated chemistry of curcumin is a reason for its diverse effects. Besides, curcumin is capable of involving several signaling pathways of survival, cellular protection, metastasis, and angiogenesis [33]. While curcumin possibly enhances chemotherapy and chemo-preventive impacts on cancer cells, it is safe and shows almost no side effects [34]. The mentioned effect is because of the different interaction of curcumin with normal tissues and cancer cells [34]. Higher cellular uptake, lower glutathione level, and active NF-κB expression in cancer cells are the reasons for the different effects of curcumin on them compared to normal cells [34].

Several studies have been concerned with the role of curcumin with a focus on its molecular targets and clinical features. For instance, curcumin effects on gastric cancer have been investigated and it is found that curcumin treatment leads to cells’ apoptosis and autophagy as well as inhibition of PI3K/Akt/mTOR signaling pathway [35]. In an open-label phase I trial, the effects of curcumin and docetaxel, a chemotherapeutic agent, co-treatment in breast cancer patients was evaluated to determine the maximal tolerable dose of the combination of dose-escalating curcumin and the standard dose of docetaxel [36]. In another study conducted on EC, curcumin treatment has been found to suppress Bcl-2 expression [37]. Co-treatment with letrozole and curcumin is reported to cause an increased inhibitory effect on tumor progression [37]. Moreover, both letrozole and curcumin can induce apoptosis [37]. Sun et al. [38] has also concluded that curcumin has the ability to downregulate MMP-2 besides inhibiting proliferation of EC cells.

## Curcumin induces apoptosis in EC cells

Apoptosis, programmed cell death, is an energy-dependent mechanism that its deregulation is a cancer hallmark [39, 40]. Although, apoptosis is necessary for vital functions of body including the turnover of normal cell, hormone-dependent atrophy, and chemical-induced cell death [40]. The changes and abnormalities in apoptosis may cause the resistance of tumors to treatments besides its undeniable roles in progression and development of tumors [39]. Moreover, a large number of anticancer drugs play their roles by targeting the apoptotic signaling pathways to initiate cancer cell death [39].

One of the processes by which curcumin exerts its anti-tumor activity is apoptosis. Curcumin leads to apoptosis in multiple cancers through involving various mechanisms. For instance, it induces apoptosis in castration-resistant prostate cancer by iron chelation [41]. In melanoma cancer cells, producing reactive oxygen species is another way that curcumin results in apoptosis [42]. Feng et al. [43] observed that curcumin led to lower expression levels of androgen receptor and beta-catenin in EC cell lines. Curcumin involves the Wnt signaling pathway to downregulate the androgen receptor which results in the inhibition of apoptosis and proliferation of EC cells [43]. In human endometrial adenocarcinoma HEC-1-A cells, it was demonstrated that high expression levels of Ets-1, a proto-oncogene, led to an increase in an anti-apoptotic protein (Bcl-2) and this up-regulation was reduced by the administration of curcumin [44]. Moreover, curcumin induced apoptosis and DNA degradation in this cell lines [44].

An investigation that used curcumin encapsulated in liposomes found that it causes alterations in the morphology of cell’s nucleus in EC cell lines (Ishikawa and HEC-1) including higher number of apoptotic chromatin condensation and fragmentation of DNA [45]. Furthermore, the results showed that liposomal curcumin considerably leads to the induction of apoptosis and inhibition of cells proliferation as well as inhibiting the expression of NF-κB, caspase-3, and MMP-9 [45]. Kumar et al. [46] reported that using curcumin loaded mixed micelles would increase the apoptotic population to 67.97% from 32.56% in free curcumin. After the treatment with curcumin chromatin condensation, pyknosis of nucleolus, and apoptotic bodies were observed which are typical characteristics of apoptosis [46]. In curcumin loaded mixed micelles, expression levels of survivin, bcl-2, PARP, and Mdr which are anti-apoptotic factors showed a significant reduction [46]. Furthermore, Kumar et al. [46] indicated that curcumin loaded mixed micelles can cause cell cycle arrest at G0/G1 phase and modulate the levels of TNF-α, IL-6 and IL-10. A paper resulted that treating EC cell lines (Ishikawa and RL95-2) with curcumin (40–50 mM) leads to a 60–80% reduction in the viability of cells [47]. While inactive caspases of cancer cells need the protein cleavage in order to become active and take part in apoptosis, cells treated with curcumin has been demonstrated cleavage of active caspase-3 [47, 48]. Curcumin-treated cells expressed less IL-6 that induced phosphorylation of STAT-3 [47]. The STAT-3 phosphorylation is linked with reduced cell viability and enhanced caspase-3 cleavage [47]. Also, curcumin treatment resulted in the inhibition of JAK-STAT signaling as well as increased SOCS-3 leading to enhanced STAT-3 phosphorylation and cell viability [47].

## Curcumin inhibitory effect on cell migration and invasion

In vivo, cancer cells’ migration is through the progressive degradation of the surrounding extracellular matrix to make migration tracks for themselves [49]. Cell migration is a critical process in cancer metastasis [50]. Several studies have shown that the two closely linked processes, invasive growth, and metastasis, are principal signs of tumor progression [51]. Severe failure of organs is a result of massive metastatic lesions which are possibly followed by a patient’s death [51]. Remarkably, significant numbers of solid tumor mortalities occur due to the cancer metastases, and the inability to treat them [49].

Curcumin has been shown to reduce the movement and invasion of EC cell lines (HEC-1B and Ishikawa) [52]. Matrix metalloproteinases (MMPs) have an undeniable function in a variety of tumor formations such as growth, invasion, and metastasis of tumors and events that occurred in early carcinogenesis [53]. MMP-2, MMP-9, and proteinase activity have been decreased by curcumin treatment [52]. Western blot assays have shown that curcumin causes a significant reduction in phosphorylated extracellular signal-regulated kinase (ERK) 1/2 [52]. Also, co-treatment of HEC-1B cells with curcumin and ERK inhibitor, U0126, leads to a suppression of cells invasiveness as well as an enhanced decrease in expressions of MMP-2 and MMP-9 [52]. Sirohi et al. [54] found that curcumin inhibits the cancer cells proliferation and tumor growth in Ishikawa cells both in vivo and in vitro. Scratch wound assay showed that curcumin inhibits the migration of Ishikawa and Hec-1B cells [54]. Besides the induction of apoptosis which is mediated by reactive oxygen species, curcumin up-regulates Slit-2 expression in Ishikawa, Hec-1B, and primary endometrial cancer cells [54]. Meanwhile, it results in down-regulation of stromal cell-derived factor-1 (SDF-1) and CXCR4 which inhibits the expressions of MMP-2 and MMP-9; therefore, curcumin reduces cell migration [54].

## Effects of curcumin on miRNAs involved in EC

MicroRNA (miRNA) which is a small, single-stranded, non-coding RNA, is present in majority of eukaryotes including humans [55]. Studies suggested that miRNAs are responsible for the regulation of at least 30% of genes that are coding proteins [55]. By binding to target mRNA, miRNA inhibits the production of proteins [55]. Since associations between different human diseases and miRNA are being found gradually, developing new therapeutics focuses on targeting the miRNA directly [56]. Significant alterations in miRNA expression have been observed in various tumor tissues and cancer cell lines which are related to multiple biological aspects such as proliferation, differentiation, and survival [57]. In several disorders and medical conditions including cancer, dysregulation of miRNA is a biomarker and serves as an oncogene or a tumor suppressor gene [57].

MiRNA-34a is one of those miRNAs which are involved and exert several roles in EC [58–61]. For instance, miR-34a is down-regulated in EC in comparison with normal tissue, and this down-regulation is linked with a poorer prognosis [60]. MiR-34a modulates the expression of MMSET which is suggested to be a pro-metastatic agent; thus, it reduces the invasion of EC cells [60]. Through down-regulating Notch1, miR-34a inhibits the proliferation, migration, invasion, and phenotypes associated with EMT in EC cells [61]. The effects of curcumin on miR-34a has been investigated in multiple cancers including gastric cancer, colorectal cancer, prostate cancer, and breast cancer [62–65]. While curcumin is shown to induce apoptosis and inhibit proliferation in gastric cancer, it is suggested that these effects may be associated with its ability to increase the expression level of miR-34a which can affect Bcl-2, CDK4, and cyclin D1 [63]. In another study, it is observed that curcumin led to an increase in miR-34a expression as well as down-regulation of β-catenin and c-myc [65]. Furthermore, curcumin anti-proliferative effects were suppressed and the β-catenin/c-myc axis was activated by inhibiting miR-34a [65].

In EC miR-21 expression is upregulated and relation is found between this miRNA and maspin which is a tumor suppressor gene [66]. In endometrioid EC cells, the upregulation of miR-21 has been observed to result in a significant reduction in the expression level of Phosphatase and tensin homolog deleted from chromosome-10 (PTEN) protein which is a tumor-suppressor gene [67]. Curcumin can reduce miR-21 [68]. Furthermore, it is suggested that curcumin plays its multiple anti-tumor properties through involving miR-21 such as proliferation, apoptosis, metastasis, and resistance to anti-cancer drugs [68]. Data shows that curcumin treatment leads to a decrease in both activity and expression of miR-21 promoter through suppressing binding of activator protein 1 [69]. Besides, curcumin induces a target of miR-21, tumor suppressor programmed cell death protein 4 (Pdcd4) [69].

Over-expression of forkhead box protein O1 (FOXO1), a down-regulated tumor suppressor in EC, has been observed to inhibit proliferation of Ishikawa cells as well as suppression of cell migration and invasion [70, 71]. It is shown that FOXO1 is significantly decreased by some miRNAs in HEC-1B cells including miR-9, miR-27, miR-96, miR-153, miR-182, miR-183, or miR-186 [71]. An investigation found that curcumin suppressed cell proliferation by miR-9 up-regulation and inhibited Wnt/β-catenin signaling in oral squamous cell carcinoma [72]. In a study on the ovarian cancer cell line, curcumin treatment led to a significant increase in miR-9 [73]. Over-expression of miR-9 increased caspase-3 cleavage and enhanced apoptosis [73]. Moreover, Akt and FOXO1 phosphorylation was reduced by both curcumin and overexpression of miR-9 [73]. Therefore, it is demonstrated that curcumin anti-tumor effects on this cancer are mostly through miR-9 up-regulation [73]. Also, another study suggests that curcumin may affect expression levels of miR-183 [74].

## Curcumin anti‐inflammatory roles in EC

Local and chronic inflammation can be a predisposing factor for development of cancer since it leads to the generation of free radicals as well as increasing COX-2 and PGE_2_; therefore, it may cause damage to DNA and proliferation of cells [75]. Furthermore, chronic inflammation may disrupt NF-κB pathway regulation which result is apoptosis suppression, inhibition of cell cycle arrest, and induction of pro-inflammatory cytokines [75]. Events that occur in the menstrual cycle are similar to inflammation mechanisms [75]. On the other hand, one of the processes which make obesity related to a higher risk of EC is inflammation [76]. Adipose tissue secretes several pro-and anti-inflammatory cytokines including tumor necrosis factor (TNF)-α, leptin, interleukin (IL)-6, C-reactive protein (CRP), and adiponectin, respectively [76]. Besides, obesity leads to higher pro-inflammatory markers and lower anti-inflammatory markers and enhances the status of chronic low-grade inflammation [76].

Curcumin has multiple effects on inflammation and obesity-associated inflammatory conditions which could be useful in treating EC. Curcumin results in modulation of TNF-α expression through making an effect on TNF-α promoter methylation status [77]. Curcumin regulates the toxic effect in adipocytes since it decreases the secretion of inflammatory cytokines which leads to a protective impact on hypoxia [77]. Also, TNF-α, COX-2, STAT, cyclinD1, and NF-ĸB signaling pathways can be inhibited by curcumin [77]. Curcumin has been found to suppress obesity-associated inflammation besides its beneficial effects on systemic inflammation, hyperglycemia, and resistance to insulin [78]. In obesity, this anti-inflammatory agent involves WAT and regulates different targets including inhibition of low-grade chronic inflammation, increasing anti-oxidant responses, and decreasing the formation of adipose tissue [78].

## Conclusions

**Fig. 1:**
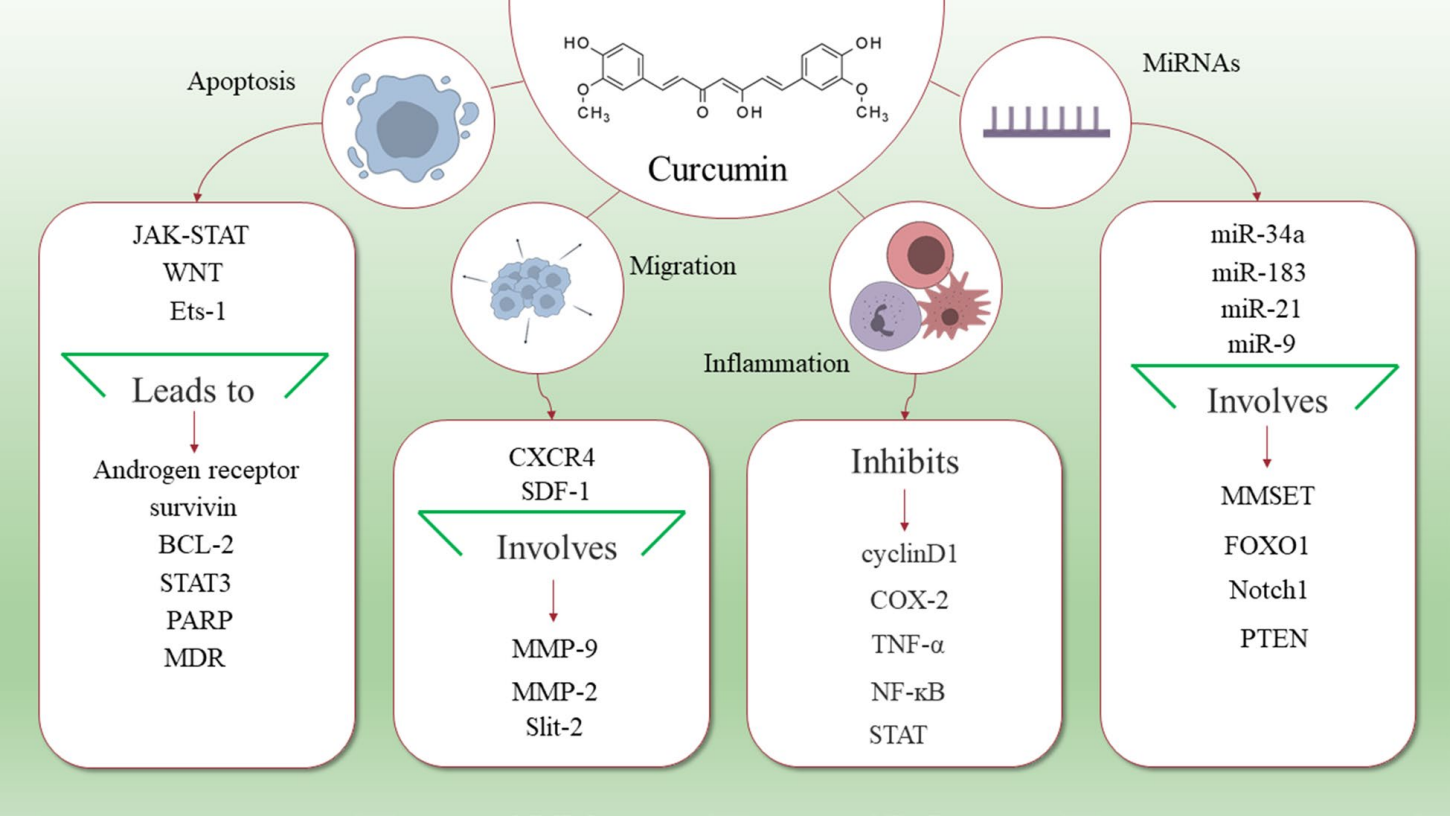
Schematic representation of curcumin targets that are useful for treating EC. As it is shown in this figure, curcumin targets a variety of molecules and signaling pathways that lead to its anti-tumor effects, including induction of apoptosis, suppressing inflammation, and preventing migration. Furthermore, some microRNAs that are targeted by curcumin may be useful in the treatment of EC

The mortality rates of EC are increasing in older patients. Furthermore, incidence rate of it is growing in the general population. A considerable number of patients show regional or distant metastasis, although more than 70% of cases are diagnosed at early stages. Therefore, considering potential therapeutic targets for treating EC is a critical step to enhance survival and life quality of the patients. Curcumin has complex chemistry and it is capable of targeting some signaling pathways. Moreover, it can interact with several intracellular and extracellular molecules. These features lead to anti-tumor effects of curcumin on various cancer cells and is useful at different stages, including prevention, treatment, and controlling the symptoms of cancers. There are studies concerned with the anti-tumor effects of curcumin in the treatment of EC (Fig. 1). Curcumin plays these roles by involving various targets, such as signaling pathways, proteins, genes, and RNAs. Induction of apoptosis, reducing inflammation, and inhibiting cell migration are the results of curcumin treatment. Furthermore, there are some miRNAs whose effects on EC have been identified and curcumin has been observed to impact on these miRNAs but in other cancers. However, to the best of our knowledge, studies about curcumin effects on EC, especially at the clinical level, are limited. Altogether, curcumin should be considered as a therapeutic target in EC and its anti-tumor effects on this cancer deserve further exploration.

## Data Availability

Not applicable.

## Abbreviations

EC: Endometrial cancer
miRNA: MicroRNA
MMP: Matrix metalloproteinase

## References

[1] Vogel H, Pelletier J (1815). Curcumin-biological and medicinal properties. J Pharma..

[2] Thomas-Eapen NE (2009). Turmeric: the intriguing yellow spice with medicinal properties. Explore.

[3] Kolev TM, Velcheva EA, Stamboliyska BA, Spiteller M (2005). DFT and experimental studies of the structure and vibrational spectra of curcumin. Int J Quantum Chem.

[4] Lee WH, Loo CY, Bebawy M, Luk F, Mason RS, Rohanizadeh R (2013). Curcumin and its derivatives: their application in neuropharmacology and neuroscience in the 21st century. Curr Neuropharmacol.

[5] Rahmani AH, Alsahli MA, Aly SM, Khan MA, Aldebasi YH (2018). Role of curcumin in disease prevention and treatment. Adv Biomed Res.

[6] Cheng AL, Hsu CH, Lin JK, Hsu MM, Ho YF, Shen TS (2001). Phase I clinical trial of curcumin, a chemopreventive agent, in patients with high-risk or pre-malignant lesions. Anticancer Res.

[7] Sharma RA, McLelland HR, Hill KA, Ireson CR, Euden SA, Manson MM (2001). Pharmacodynamic and pharmacokinetic study of oral Curcuma extract in patients with colorectal cancer. Clin Cancer Res.

[8] Sanphui P, Goud NR, Khandavilli UB, Bhanoth S, Nangia A (2011). New polymorphs of curcumin. Chem Commun.

[9] Aggarwal BB, Sung B (2009). Pharmacological basis for the role of curcumin in chronic diseases: an age-old spice with modern targets. Trends Pharmacol Sci.

[10] Maheshwari RK, Singh AK, Gaddipati J, Srimal RC (2006). Multiple biological activities of curcumin: a short review. Life Sci.

[11] Moore K, Brewer MA (2017). Endometrial cancer: is this a new disease?. Am Soc Clin Oncol Educ Book.

[12] Malpica A, Euscher ED, Hecht JL, Ali-Fehmi R, Quick CM, Singh N (2019). Endometrial carcinoma, grossing and processing issues: recommendations of the international society of gynecologic pathologists. Int J Gynecol Pathol.

[13] Bokhman JV (1983). Two pathogenetic types of endometrial carcinoma. Gynecol Oncol.

[14] Samarnthai N, Hall K, Yeh C-K (2010). Molecular profiling of endometrial malignancies. Obstet Gynecol Int.

[15] Arend RC, Jones BA, Martinez A, Goodfellow P (2018). Endometrial cancer: molecular markers and management of advanced stage disease. Gynecol Oncol.

[16] Gupta D (2017). Clinical behavior and treatment of endometrial cancer. Adv Exp Med Biol.

[17] Haldorsen IS, Salvesen HB (2016). What is the best preoperative imaging for endometrial cancer?. Curr Oncol Rep.

[18] Larson DM, Connor GP, Broste SK, Krawisz BR, Johnson KK (1996). Prognostic significance of gross myometrial invasion with endometrial cancer. Obstet Gynecol.

[19] Boronow RC, Morrow CP, Creasman WT, Disaia PJ, Silverberg SG, Miller A (1984). Surgical staging in endometrial cancer: clinical-pathologic findings of a prospective study. Obstet Gynecol.

[20] Mittal P, Klingler-Hoffmann M, Arentz G, Zhang C, Kaur G, Oehler MK (2016). Proteomics of endometrial cancer diagnosis, treatment, and prognosis. Proteomics Clin Appl.

[21] Ali AT (2013). Risk factors for endometrial cancer. Ceska Gynekol.

[22] Sorosky JI (2012). Endometrial cancer. Obstet Gynecol.

[23] Sun KX, Chen Y, Chen S, Liu BL, Feng MX, Zong ZH (2016). The correlation between microRNA490-3p and TGFalpha in endometrial carcinoma tumorigenesis and progression. Oncotarget.

[24] Chaudhry P, Asselin E (2009). Resistance to chemotherapy and hormone therapy in endometrial cancer. Endocr Relat Cancer.

[25] Yeramian A, Garcia V, Bergada L, Domingo M, Santacana M, Valls J (2016). Bioluminescence imaging to monitor the effects of the Hsp90 inhibitor NVP-AUY922 on NF-kappaB pathway in endometrial cancer. Mol Imaging Biol.

[26] Shanmugam MK, Rane G, Kanchi MM, Arfuso F, Chinnathambi A, Zayed ME (2015). The multifaceted role of curcumin in cancer prevention and treatment. Molecules.

[27] Anand P, Sundaram C, Jhurani S, Kunnumakkara AB, Aggarwal BB (2008). Curcumin and cancer: an “old-age” disease with an “age-old” solution. Cancer Lett.

[28] Banik U, Parasuraman S, Adhikary AK, Othman NH (2017). Curcumin: the spicy modulator of breast carcinogenesis. J Exp Clin Cancer Res.

[29] Jordan BC, Mock CD, Thilagavathi R, Selvam C (2016). Molecular mechanisms of curcumin and its semisynthetic analogues in prostate cancer prevention and treatment. Life Sci.

[30] Lelli D, Pedone C, Majeed M, Sahebkar A (2017). Curcumin and lung cancer: the role of microRNAs. Curr Pharm Des.

[31] Lelli D, Pedone C, Sahebkar A (2017). Curcumin and treatment of melanoma: the potential role of microRNAs. Biomed Pharmacother.

[32] Simental-Mendia LE, Caraglia M, Majeed M, Sahebkar A (2017). Impact of curcumin on the regulation of microRNAs in colorectal cancer. Expert Rev Gastroenterol Hepatol.

[33] Hatcher H, Planalp R, Cho J, Torti FM, Torti SV (2008). Curcumin: from ancient medicine to current clinical trials. Cell Mol Life Sci.

[34] Allegra A, Innao V, Russo S, Gerace D, Alonci A, Musolino C (2017). Anticancer activity of curcumin and its analogues: preclinical and clinical studies. Cancer Invest.

[35] Li W, Zhou Y, Yang J, Li H, Zhang H, Zheng P (2017). Curcumin induces apoptotic cell death and protective autophagy in human gastric cancer cells. Oncol Rep.

[36] Gupta SC, Patchva S, Aggarwal BB (2013). Therapeutic roles of curcumin: lessons learned from clinical trials. AAPS J.

[37] Liang YJ, Hao Q, Wu YZ, Wang QL, Wang JD, Hu YL (2009). Aromatase inhibitor letrozole in synergy with curcumin in the inhibition of xenografted endometrial carcinoma growth. Int J Gynecol Cancer.

[38] Sun MX, Yu F, Gong ML, Fan GL, Liu CX (2018). Effects of curcumin on the role of MMP-2 in endometrial cancer cell proliferation and invasion. Eur Rev Med Pharmacol Sci.

[39] Pistritto G, Trisciuoglio D, Ceci C, Garufi A, D’Orazi G (2016). Apoptosis as anticancer mechanism: function and dysfunction of its modulators and targeted therapeutic strategies. Aging.

[40] Elmore S (2007). Apoptosis: a review of programmed cell death. Toxicol Pathol.

[41] Yang C, Ma X, Wang Z, Zeng X, Hu Z, Ye Z (2017). Curcumin induces apoptosis and protective autophagy in castration-resistant prostate cancer cells through iron chelation. Drug Des Devel Ther.

[42] Kocyigit A, Guler EM (2017). Curcumin induce DNA damage and apoptosis through generation of reactive oxygen species and reducing mitochondrial membrane potential in melanoma cancer cells. Cell Mol Biol (Noisy-le-grand).

[43] Feng W, Yang CX, Zhang L, Fang Y, Yan M (2014). Curcumin promotes the apoptosis of human endometrial carcinoma cells by downregulating the expression of androgen receptor through Wnt signal pathway. Eur J Gynaecol Oncol.

[44] Yu Z, Shah DM (2007). Curcumin down-regulates Ets-1 and Bcl-2 expression in human endometrial carcinoma HEC-1-A cells. Gynecol Oncol.

[45] Xu H, Gong Z, Zhou S, Yang S, Wang D, Chen X (2018). Liposomal curcumin targeting endometrial cancer through the NF-kappaB pathway. Cell Physiol Biochem.

[46] Kumar A, Sirohi VK, Anum F, Singh PK, Gupta K, Gupta D (2017). Enhanced apoptosis, survivin down-regulation and assisted immunochemotherapy by curcumin loaded amphiphilic mixed micelles for subjugating endometrial cancer. Nanomedicine.

[47] Saydmohammed M, Joseph D, Syed V (2010). Curcumin suppresses constitutive activation of STAT-3 by up-regulating protein inhibitor of activated STAT-3 (PIAS-3) in ovarian and endometrial cancer cells. J Cell Biochem.

[48] Hensley P, Mishra M, Kyprianou N (2013). Targeting caspases in cancer therapeutics. Biol Chem.

[49] Paul CD, Mistriotis P, Konstantopoulos K (2017). Cancer cell motility: lessons from migration in confined spaces. Nat Rev Cancer.

[50] Wirtz D, Konstantopoulos K, Searson PC (2011). The physics of cancer: the role of physical interactions and mechanical forces in metastasis. Nat Rev Cancer.

[51] Krakhmal NV, Zavyalova MV, Denisov EV, Vtorushin SV, Perelmuter VM (2015). Cancer invasion: patterns and mechanisms. Acta Naturae.

[52] Chen Q, Gao Q, Chen K, Wang Y, Chen L, Li XU (2015). Curcumin suppresses migration and invasion of human endometrial carcinoma cells. Oncol Lett.

[53] Nabeshima K, Inoue T, Shimao Y, Sameshima T (2002). Matrix metalloproteinases in tumor invasion: role for cell migration. Pathol Int.

[54] Sirohi VK, Popli P, Sankhwar P, Kaushal JB, Gupta K, Manohar M (2017). Curcumin exhibits anti-tumor effect and attenuates cellular migration via Slit-2 mediated down-regulation of SDF-1 and CXCR4 in endometrial adenocarcinoma cells. J Nutr Biochem.

[55] Macfarlane LA, Murphy PR (2010). MicroRNA: biogenesis, function and role in cancer. Curr Genomics.

[56] Hammond SM (2015). An overview of microRNAs. Adv Drug Deliv Rev.

[57] Ji W, Sun B, Su C (2017). Targeting microRNAs in cancer gene therapy. Genes..

[58] Corrado G, Laquintana V, Loria R, Carosi M, de Salvo L, Sperduti I (2018). Endometrial cancer prognosis correlates with the expression of L1CAM and miR34a biomarkers. J Exp Clin Cancer Res.

[59] Choi YS, Lee KE (2015). The significance of miR-34a expression in endometrial carcinogenesis: correlation with expression of p16 and Ki-67 proteins in endometrial cancers. J Cancer Prev.

[60] Dong P, Xiong Y, Yue J, Hanley SJB, Watari H (2018). miR-34a, miR-424 and miR-513 inhibit MMSET expression to repress endometrial cancer cell invasion and sphere formation. Oncotarget.

[61] Wang Z, Wang W, Huang K, Wang Y, Li J, Yang X (2017). MicroRNA-34a inhibits cells proliferation and invasion by downregulating Notch1 in endometrial cancer. Oncotarget.

[62] Guo J, Li W, Shi H, Xie X, Li L, Tang H (2013). Synergistic effects of curcumin with emodin against the proliferation and invasion of breast cancer cells through upregulation of miR-34a. Mol Cell Biochem.

[63] Sun C, Zhang S, Liu C, Liu X (2019). Curcumin promoted miR-34a expression and suppressed proliferation of gastric cancer cells. Cancer Biother Radiopharm..

[64] Toden S, Okugawa Y, Buhrmann C, Nattamai D, Anguiano E, Baldwin N (2015). Novel evidence for curcumin and Boswellic acid-induced chemoprevention through regulation of miR-34a and miR-27a in colorectal cancer. Cancer Prev Res.

[65] Zhu M, Zheng Z, Huang J, Ma X, Huang C, Wu R (2019). Modulation of miR-34a in curcumin-induced antiproliferation of prostate cancer cells. J Cell Biochem.

[66] Torres A, Torres K, Paszkowski T, Radej S, Staskiewicz GJ, Ceccaroni M (2011). Highly increased maspin expression corresponds with up-regulation of miR-21 in endometrial cancer: a preliminary report. Int J Gynecol Cancer.

[67] Qin X, Yan L, Zhao X, Li C, Fu Y (2012). microRNA-21 overexpression contributes to cell proliferation by targeting PTEN in endometrioid endometrial cancer. Oncol Lett.

[68] Chen J, Xu T, Chen C (2015). The critical roles of miR-21 in anti-cancer effects of curcumin. Ann Transl Med.

[69] Mudduluru G, George-William JN, Muppala S, Asangani IA, Kumarswamy R, Nelson LD (2011). Curcumin regulates miR-21 expression and inhibits invasion and metastasis in colorectal cancer. Biosci Rep.

[70] Zhang Y, Zhang L, Sun H, Lv Q, Qiu C, Che X (2017). Forkhead transcription factor 1 inhibits endometrial cancer cell proliferation via sterol regulatory element-binding protein 1. Oncol Lett.

[71] Myatt SS, Wang J, Monteiro LJ, Christian M, Ho KK, Fusi L (2010). Definition of microRNAs that repress expression of the tumor suppressor gene FOXO1 in endometrial cancer. Cancer Res.

[72] Xiao C, Wang L, Zhu L, Zhang C, Zhou J (2014). Curcumin inhibits oral squamous cell carcinoma SCC-9 cells proliferation by regulating miR-9 expression. Biochem Biophys Res Commun.

[73] Zhao SF, Zhang X, Zhang XJ, Shi XQ, Yu ZJ, Kan QC (2014). Induction of microRNA-9 mediates cytotoxicity of curcumin against SKOV3 ovarian cancer cells. Asian Pac J Cancer Prev.

[74] Teiten MH, Gaigneaux A, Chateauvieux S, Billing AM, Planchon S, Fack F (2012). Identification of differentially expressed proteins in curcumin-treated prostate cancer cell lines. OMICS.

[75] Modugno F, Ness RB, Chen C, Weiss NS (2005). Inflammation and endometrial cancer: a hypothesis. Cancer Epidemiol Biomarkers Prev.

[76] Shaw E, Farris M, McNeil J, Friedenreich C (2016). Obesity and endometrial cancer. Recent Results Cancer Res.

[77] Ghosh S, Banerjee S, Sil PC (2015). The beneficial role of curcumin on inflammation, diabetes and neurodegenerative disease: a recent update. Food Chem Toxicol.

[78] Bradford PG (2013). Curcumin and obesity. Biofactors.

